# “Unraveling the Diagnostic Dilemma: Unusual Presentation of Huntington’s Disease with Predominant Psychiatric Symptoms and Late-Onset Motor Manifestations”

**DOI:** 10.1192/j.eurpsy.2024.994

**Published:** 2024-08-27

**Authors:** M. I. M. M. N. Ibrahim, M. Iderapalli

**Affiliations:** Essex Partnership University NHS Foundation Trust, Basildon, United Kingdom

## Abstract

**Introduction:**

Huntington’s Disease is a neurodegenerative disease inherited in an autosomal dominant fashion.The underlying genetic defect is unstable CAG trinucleotide repeat expansion with a repeat length longer than 36 resulting in pathological aggregation of abnormal protein causing cell death.

The clinical symptoms encompass 3 main domains-motor,cognitive and psychiatric.The psychiatric symptoms often in atypical form appear decades before other symptoms causing significant impact on patient’s functioning and quality of life.

Here, we discuss an unusual presentation of Huntington’s Disease causing diagnostic dilemma.

**Objectives:**

Case report discussing the unusual presentation of Huntington’s Disease.

**Methods:**

Case: Mr X is a 61 year old Caucasian male.He had an uneventful birth and early childhood attaining milestones appropriately. He experienced childhood adversity in the form of sexual abuse between ages 2-14 years. His mental health difficulties started following sexual abuse when he attempted to end his life by hanging and overdosing at age 15. He got married twice, both of which broke down. There is a history of significant alcohol abuse between ages 40-50. Following this, he had a myocardial infarction and a stroke requiring stenting.

He presented to Psychiatric Outpatient Services in 2011 with auditory hallucinations, social anxiety with panic attacks, OCD type rituals, claustrophobia and feeling hot all the time. He was started on an antipsychotic medication for psychosis ,but clinically deteriorated. He started having anger outbursts, marching on the spot ,and head banging. He was diagnosed with Huntington’s Chorea in 2021 after he had developed chorea. He currently has low mood and is head banging for hours.

**Results:**

Psychiatric symptoms in HD can span a variety of domains but most common are symptoms of frontal lobe dysfunction-disinhibition, poor attention, irritability, impulsivity and personality change. Apathy, emotional blandness and social withdrawal are also prominent features.

Mr X had strong family history of Paranoid Schizophrenia (aunt and cousin).There was no family history of HD. His mental health problems started early in life with DSH, Depression and Harmful use of Alcohol. He presented predominantly with psychotic symptoms like auditory hallucinations, social anxiety, paranoia. Motor symptoms started late which he incorporated into voluntary movements like head banging which made it difficult to differentiate from deliberate self harm.

**Conclusions:**

Psychiatric symptoms constitute the core of HD. Studies have shown that though depression and personality change are typical of HD, there are number of other psychiatric symptoms that can impair quality of life. Early diagnosis and treatment of these symptoms will help patients and families to cope better with severe symptoms of this progressive disease.

**Disclosure of Interest:**

None Declared

